# Association between dopamine genes, adiposity, food addiction, and eating behavior in Chilean adult

**DOI:** 10.3389/fnut.2024.1466384

**Published:** 2024-09-25

**Authors:** Nicole Luengo, Gary S. Goldfield, Ana M. Obregón

**Affiliations:** ^1^Escuela de Nutrición y Dietética, Facultad de Ciencias para el cuidado de la Salud, Universidad San Sebastián, Concepción, Chile; ^2^Children’s Hospital of Eastern Ontario Research Institute, Ottawa, ON, Canada

**Keywords:** food addiction, polymorphism, eating behavior, dopamine, obesity

## Abstract

**Background:**

A frequent consumption of high sugar/fat foods can affect dopamine signaling in the brain and cause sustained stimulation of the reward system. It has been hypothesized that a hypodopaminergic trait results in an individual overeating in order to increase brain DA. Genetic variants in this route have been connected with addiction and eating behaviors. Most studies focus on a specific SNP, and few studies have used multilocus genetic scores, which quantify genetic risk on a continuum.

**Aim:**

To assess the relationship between multilocus genetic scores based on multiple gene variants in the dopaminergic pathway and measurements of anthropometry, eating behavior, food reinforcement, and food addiction (FA) in Chilean adults.

**Methods:**

We recruited 221 Chilean adults for a cross-sectional study. A standard anthropometric measurement procedure was followed and eating behavior was examined using the Three Factor Eating questionnaire (TFEQ), Food Reinforcement Value Questionnaire (FRVQ), Yale Food Addiction Scale (YFAS) and 24-h diet recall. Multilocus genetic scores were calculated using TaqMan assays (rs1800497-rs1799732-rs6277-rs4680).

**Results:**

No differences were found in the entire sample for anthropometric measurements, by MLGS. We found that participants with a score ≥ 2.0 in the MLGS showed higher food choices on the RVFQ and lower energy intake in protein, lipids, SAFA, MUFA, PUFA, dietary cholesterol, omega-3 and Omega-6 fatty acids in the 24-h recall (*p* < 0.05). Stratified by nutritional condition, the group with obesity had inferior scores on cognitive restriction, greater scores on uncontrolled eating, emotional eating, and responding to palatable food in the RVFQ. Also, in subjects with obesity, there was more food addiction in the group scoring “MLGS ≥2.0 or low dopamine signaling” (53%), compared to the group scored “MLGS <2.0 or high dopamine signaling” (23%) (*p*-value; 0.05). Emotional Eating scores correlated positively with MLGS in subjects with obesity.

**Conclusion:**

In adults with obesity, the MLGS of the dopamine pathway, reflecting hypodopaminergic signaling, was associated with greater scores on food addiction and altered eating behavior traits.

## Introduction

Obesity is a worldwide problem (1). It could be due to interactions among environmental and genetic factor (2, 3). Considering the 2016 levels of childhood obesity in the USA, simulated growth trajectories suggest 57% of today’s children will be obese by 35 (1). Like many other high-income and developing countries, Chile’s overweight and obesity rate is rapidly increasing, most notably among preschool and school-aged children. The last National Health Survey inform that 74% of the adult population had excess. According to the research, 27.6% of adolescents (15–19 years) are overweight, 12.2% are obese, and 1% are severely obese (4). One of the question that scientists face in this field is related to eating behavior, overeating and food craving with addictive behavior (5, 6). Studies has established that frequent ingestion of high sugar/fat food can produce changes in brain dopamine signaling (7, 8). This can result in abnormally sustained reward system stimulation (9). Food is rewarding, in part, through activation of the mesolimbic dopamine (DA) pathway. A high sugar and fat content in some foods can act like drugs, causing compulsive eating and loss of control (7). There is an emerging literature investigating dopamine genes in relationship to addictive and compulsive appetitive behaviors (10, 11).

A recent 2016 genome-wide association study (GWAS) of 9,314 females of European ancestry who were identified as having food addiction by the modified YFAS (Yale Food Addiction Scale) did not identify a significant association with any single nucleotide polymorphisms (SNPs) or genes implicated in drug addiction (12). Nevertheless, a 2015 study of neurogenetic and neuroimaging evidence for a theoretical model of dopaminergic influences to obesity, found a collection of research involving an association between obesity and genetic variants in DA receptors genes for DA receptors 2, 3, 4 (DRD2, DRD3, and DRD4), dopamine transporter 1 (DAT1) and genes for enzymes implicated with dopamine degradation—catechol-o-methyltransferase (COMT) and monoamine oxidase isomers A and B (13). Although there are no evidences for the involvement of common variants near DRD2 gene from genome-wide association study, there are several association studies that have reported a possible involvement of DRD2 variants in eating behavior traits. In this field, we previously showed that rs1800497 was not associated with food addiction, but in obese female A1 carriers was associated with scores of emotional eating and snacking reinforcement (14). We then looked for the relation between the bilocus genetic profile (rs1799732 + rs1800497) and food addiction in the same sample, showing no association (15). Considering that only two variants account for a minimal percentage of phenotypical variation we decide to explore other methods.

In genetics, to predict an individual’s risk of developing a particular trait or disease based on genetic data, certain tools are often used. Some studies use a Polygenic Risk Score (PRS) which is a numerical representation of the estimated effects of many genetic variants across the genome on an individual’s phenotype, particularly complex traits. A PRS represents an individual’s genetic predisposition to a trait or disease by integrating the cumulative impact of numerous small-effect variants, which are weighted based on their effect sizes in genome-wide association studies. An alternative approach, is the Multilocus Genetic Score (MLGS), which focuses on a specific set of genetic loci believed to contribute to a particular phenotype or disease. This method aggregates the effects of these loci, not necessarily derived from a genome-wide association studies. The MLGS can be seen as a more targeted approach, often reflecting the additive risk from a limited number of genes hypothesized to be involved in a specific biological pathway or trait.

Using the concept of candidate gene clustering, a multilocus genetic score was developed established on polymorphisms in multiple reward markers related to changes in dopamine transmission in the brain. Previously, Nikolova et al. (16) reported that MLGS was associated with higher DA signaling, predicting an increase in reward-related activity in the ventral striatum. According to this finding, a multilocus profiling method could capture the accumulative effect of genetic variants whose single effects might be undetected in small samples. After that, Davis et al. (17) showed that MLGP scores were higher in people with YFAS and that binge eating, cravings, and emotional overeating were positively correlated. In a similar manner, Yokum et al. found that participants with a greater number of alleles associated with DA signaling capacity, showed greater weight gain than those with fewer risk alleles (18).

Given this evidence, the aim if this study was to assess the relationship between multilocus genetic scores based on multiple gene variants in the dopaminergic pathway and measurements of anthropometry, eating behavior, food reinforcement, and food addiction (FA) in Chilean adults. We hypothesized that higher multilocus genetic score would exhibit higher adiposity, scores of unhealthy eating behavior and food addiction compared with lower scores.

## Materials and methods

A cross- sectional study was developed between January 2016 and March 2017. The inclusion criteria were (i) subjects aged ≥18 years; without consumption of medications that affected body weight, and without treatment to lose weight. The exclusion criteria were (i) Patients with diseases such as genetic syndromes, pregnant women, individuals with associated diagnoses of cardiovascular, liver, kidney or cancer; and (ii) other pathologies that require dietary restrictions.

A convenience sample was recruited involving 221 adults (74% female, 18–54 years old), 43.8% with obesity, 11.3% overweight, and 44.8% normal weight. Participants were recruited through a variety of sources in the community, including posters, on campus at Universidad San Sebastian, recreational and community centers, as well as online advising on the website.^1^ Informed consent was achieved from all subjects and laboratory tests occurred at San Sebastian University. The study was approved by the Research and Scientific Ethics Committee of San Sebastian University (#48–2021-20). The protocol was conducted in agreement with the Declaration of Helsinki research ethics guidelines.

### Anthropometry

We measured height, weight and waist circumference without shoes, using a weight scale (Seca 700) with a stadiometer included (100 gr. and 0.5 cm sensitivity) (19).

Based on criteria established by the World Health Organization (20), BMI cut-offs were used to determine weight status. Subjects were classified as normal-weight, overweight or obese according to their BMI values (≥ 24.9 Kg/m^2^, ≥ 25.0–29.9 Kg/m^2^, or ≥ 30.0 Kg/m^2^, respectively). After an overnight fast, the body composition was assessed using bioelectrical impedance, based on the manufacturer’s instructions using a Tanita TBF-300MA (Tanita Corporation, Tokyo, Japan).

Eating behavior: four questionnaires validated were used:

(1) *Three Factor Eating Behavior Questionnaire*: Using this instrument, 18 items are assessed and three components of eating behavior are evaluated. These components are: cognitive restraint (CR), emotional eating (EE), and uncontrolled eating (UE). Using a 4-point Likert scale, subjects rate their level of agreement on each item. Each subscale’s score was calculated by summing individual raw scores and dividing them by the number of items in that subscale (21). A Cronbach-alpha value of 0.60–0.88 was found for all subscales in the present study, suggesting moderate-to-strong internal consistency (22).
(2) *Food Reinforcement Value Questionnaire (FRVQ)*: A 12-item questionnaire assesses the relative reinforcing value of food compared to an alternative reinforcer. Using this task, we assessed subjects’ motivation to work toward obtaining either their most preferred snack food or their highest rated healthy alternative (fruits/vegetables). In this paradigm, work was defined as the number of button presses, with more button presses indicating a higher level of reinforcement. First, a fixed ratio schedule was applied, which required subjects to press the joystick button 20 times to access either snack food or fruit/vegetables. Among the remaining items, the reinforcement schedule for gaining access to preferred snack food increased by 20 button presses to a maximum of 240 button presses for item 12. In contrast, the reinforcement program for preferred fruits/vegetables remained the same. The amount of button presses associated with snack food choices denoted the relative reinforcing value of snack food. Based on the food choices made, it was expressed as a percentage. The validity of this tool has been established against a gold-standard in adults (23), and suggests good predictive validity since they predict weight gain over time (24).
(3) *24-h diet recall*: A staff of nutritionists evaluated each participant’s total energy intake, macronutrient, fiber, saturated, monounsaturated, and polyunsaturated fat intake as well as total n-6 and n-3 fatty acids using 24-h dietary recalls on days randomly select. This survey estimate energy and nutrient intake based on exhaustive food descriptions, comprising ingredient names, preparations, portions, and brand (25). Using Food Processor w/PS 10.15, 24-h recalls were analyzed for each patient.
(4) *Yale Food Addiction Scale (YFAS) (First version)*: In accordance with the DSM-IV criteria for substance dependence, a 25-item questionnaire has been developed to assess symptoms of dependence on highly palatable foods (e.g., foods high in fats and/or carbohydrates). The YFAS requires the simultaneous existence of elevated clinically levels of distress for the food addiction diagnosis to be made. Additionally, food addiction symptoms were continuously assessed, with higher scores indicating increased susceptibility. According to Obregón et al., this instrument has been validated in Chilean adults (26), following the original validation (27).

### Collection of biologic samples

A registered nurse obtain blood samples in an EDTA-coated tube of 4 mL for molecular analysis, after an overnight fast using a standard vacuum system protocol. We collected blood. After centrifuging the EDTA-coated tube at 3,300 rpm for 10 min at room temperature, plasma was separated from buffy coat and red blood cells. According to the manufacturer’s instructions, DNA was extracted from each blood sample using the QIAGEN QIAamp DNA blood mini kit #51104 (28).

### Molecular genotyping

We choose common genetic variants near DRD2 that have been previously associated in several studies with dopamine pathway and eating behavior (16–18).

Genetic variant rs1800497: PCR-RFLP was used previously to assess this variant (14). In order to determine if a given allele was present or absent, the expected sizes of the PCR products were determined: one band of 307 bp was observed for homozygous A1/A1, three bands were observed for heterozygotes of A1/A2, 307 bp, 177 bp, and 127 bp, and two bands were observed for homozygotes of A2/A2 with expected sizes of 177 base pairs and 127 base pairs.

#### Genetic variants rs1799732, rs4680, rs6277

These variants were identified using a predesigned Taqman assay ID C_33641686_10 (Applied Biosystems) using a QuantStudioTM 3 Real-Time PCR System. For rs1799732 (Homozygous G/G, heterozygous G/Del, and homozygous Del/Del genotype); for rs4680 (Homozygous AA, heterozygous A/G, and homozygous GG) and for rs6277 (Homozygous CC, heterozygous C/T, and homozygous TT) genotype groups were determine.

#### Multilocus genetic score

We estimated individually Multilocus genetic scores using 4 genetic variants of the dopaminergic system, using a similar approach as other groups (17). There was a score of 1 for genotypes associated with low DA signaling, a score of 0 for genotypes associated with high DA signaling, and a score of 0.5 for intermediate heterozygotes. A score of 1 (“low dopaminergic signaling”) was assigned to TaqIA A1/A1, DRD2-141C Ins/Ins carriers, rs6277 (C957T; T-allele) and rs4680 COMT Met/Met genotypes. A score of 0 (“high dopaminergic signaling”), was assigned to TaqIA A2/A2, DRD2-141C Ins/Del and Del/Del carriers, rs6277 (C957T; C-allele), COMT Val/Val genotypes. Finally, a score of 0.5 (“intermediate dopaminergic signaling”) was given to TaqIA A1/A2 and COMT Met/Val genotypes. The scores were added to build a multilocus genetic score. The global score at each locus will be 0–1, and for the total path a score of 0–4 (Table 1) (29).

**Table 1:** Genotypic frequency of the SNPs studied and putatively functional association.

Gene	ID	Genotypic frequency	Functional association
ANKK1 (Taq1A C > T)	rs1800497	A1A1 21 (9.9%) A1A2 71 (33.6%) A2A2 119 (56.0%)	A1 allele or T-allele associated with reduced D2 receptor binding affinity
DRD2 -141C Ins/del	rs1799732	GG 155 (73.1%) G/del 54 (25.4%) Del/del 3 (1.42%)	The Del-allele has been associated with significantly less promoter activity and protein expression of DRD2
DRD2 C957T	rs6277 C > T	TT 76 (37.2%) CT 99 (48.5%) CC 29 (14.2%)	T allele associated with alteration in receptor binding affinity and thereby in stratial dopamine levels
COMT Val158Met	rs4680	Met/Met 35 (16.9%) Met/Val 95 (46.2%) Val/Val 76 (36.8%)	Met allele associated with a reduction in DA catabolism and therefore higher DA levels

#### Data analysis

We developed a descriptive analysis of the sample (mean or median and standard deviation). Genotype and allele frequencies were determined. Also the Hardy–Weinberg equilibrium was estimated using the goodness-of-fit X2 test. An examination of differences and associations between groups was conducted using non-parametric statistics (Mann–Whitney and Kruskal-Wallis tests), including a sex-specific analysis. Data were examined with STATA 14.0 software. In order to assess the association between the MLGS and anthropometrics and eating behavior variables, the MLGS was dichotomized into two groups (MLGS <2.0 and MLGS ≥2.0).

## Results

### Association between MLGS and anthropometric measurements

A total of 204 participants were completely genotypes in the sample. Table 1 presents the genotypic frequencies of genetic variants. All variants meet the Hardy–Weinberg equilibrium. Table 2 presents the frequency of multilocus genetic scores in our sample. The number of participants without risk alleles was only 2/204 (0.98%). 61% of the sample had a score of two or higher. 0.49% of the sample was homozygous for the four polymorphisms examined. To determine if any association existed between the MLGS and anthropometrics and eating behavior variables, the MLGS was dichotomized into two groups, MLGS <2.0 and MLGS ≥2.0. Table 3 shows that there were no differences in the entire sample for anthropometric measurements.

**Table 2:** Frequency of multilocus genetic score in Chilean university students.

Frequency multilocus genetic score									
	0 *n* = 2	0.5 *n* = 7	1 *n* = 31	1.5 *n* = 39	2 *n* = 53	2.5 *n* = 30	3.0 *n* = 23	3.5 *n* = 18	4.0 *n* = 1
Total	0.98%	3.45%	15.2%	19.2%	25.9%	14.7%	11.2%	8.8%	0.49%

Individual genetic profile scores represent the sum of “high” DA genotypes across two functional polymorphic loci. “High” genotypes received a score of 1, “low” genotypes a score of 0, and “intermediate” genotypes a score of 0.5. For example, the genetic profile score for an individual with the following 2 polymorphisms, DRD2-141C Ins/Ins and DRD2 Taq1A (0 + 0).

**Table 3:** Anthropometric measurements by Multilocus genetic score MLGS (rs1799732, rs6277, rs4680, rs1800497).

	Multilocus genetic score											
	All			Normal-weight			Over-weight			Obesity		
	<score 2 (*n* = 132)	≥score 2 (*n* = 72)	*p*-value	< score 2 (*n* = 56)	≥score 2 (*n* = 35)	*p*-value	< score 2 (*n* = 15)	≥score 2 (*n* = 9)	*p*-value	< score 2 (*n* = 61)	≥score 2 (*n* = 28)	*p*-value
Age (years)	24.9 ± 4.6	24.7 ± 5.9	0.67	23.6 ± 3.5	22.9 ± 4.6	0.64	22.5 ± 2.1	24.1 ± 2.9	0.17	26.6 ± 5.2	27.0 ± 7.3	0.98
Weight at Birth (gr)	3428.9 ± 594.6	3414.8 ± 656.2	0.80	3397.1 ± 486.0	3426.2 ± 551.7	0.22	3464.2 ± 589.0	2986.4 ± 672.7	0.08	3449.5 ± 687.5	3538.2 ± 733.4	0.69
Height at birth (cm)	50.9 ± 2.4	49.6 ± 3.3	0.48	50.3 ± 2.0	49.6 ± 2.3	0.16	49.1 ± 2.5	49.1 ± 3.2	0.58	50.1 ± 2.8	49.7 ± 4.3	0.90
Weight (kg)	75.8 ± 18.1	73.4 ± 16.4	0.40	59.9 ± 7.0	60.3 ± 5.9	0.69	72.0 ± 11.0	71.2 ± 8.9	0.65	91.3 ± 12.7	90.4 ± 10.9	0.60
Height (mts)	1.64 ± 0.08	1.63 ± 0.08	0.31	1.64 ± 0.07	1.62 ± 0.07	0.27	1.64 ± 0.10	1.62 ± 0.10	0.85	1.64 ± 0.08	1.63 ± 0.08	0.59
Body mass index (kg/mt^2^)	28.1 ± 6.3	27.7 ± 5.7	0.85	22.2 ± 1.6	22.8 ± 1.3	0.07	26.8 ± 2.9	27.0 ± 1,2	0.74	33.8 ± 4.0	34.0 ± 3.5	0.98
Waist to height ratio	0.55 ± 0.1	0.55 ± 0.09	0.92	0.46 ± 0.04	0.48 ± 0.04	0.12	0.53 ± 0.07	0.54 ± 0.03	0.83	0.64 ± 0.08	0.64 ± 0.07	0.96
Abdominal circumference (cm)	90.4 ± 17.0	88.8 ± 14.6	0.58	75.7 ± 6.6	77.3 ± 6.3	0.30	87.2 ± 10.3	87.4 ± 5.5	0.92	104.9 ± 11.9	103.6 ± 10.2	0.68
Body fat %	32.0 ± 10.9	31.9 ± 10.0	0.99	23.7 ± 6.0	25.3 ± 6.3	0.13	27.5 ± 10.3	29.4 ± 8.2	0.83	40.8 ± 7.5	41.1 ± 6.8	0.91

^*^Significant differences were analyzed with the nonparametric Mann–Whitney by MLGP group.

*p < 0.05; **p < 0.001.

### Association between MLGS and eating behaviors measurements

Table 4 shows the results for eating behavior variables. In the entire sample subjects scoring MLGS ≥2.0 (*Low dopamine signaling*) showed no difference in the Eating behavior scores. We found higher % of food choice in the RVFQ and lower energy, protein, lipids, SAFA, MUFA, PUFA, dietary cholesterol, omega-3 and Omega-6 fatty acids in the 24-h recall (*p* < 0.05).

**Table 4:** Eating behavior by multilocus genetic score MLGS (rs1799732, rs6277, rs4680, rs1800497).

	Multilocus genetic score											
	All			Normal-weight			Over-weight			Obesity		
	<score 2 (*n* = 132)	≥score 2 (*n* = 72)	*p*-value	< score 2 (*n* = 56)	≥score 2 (*n* = 35)	*p*-value	< score 2 (*n* = 15)	≥score 2 (*n* = 9)	*p*-value	< score 2 (*n* = 61)	≥score 2 (*n* = 28)	*p*-value
TFEQ												
Cognitive restraint	2.3 ± 0.58	2.3 ± 0.67	0.95	2.2 ± 0.65	2.5 ± 0.6	0.07	2.28 ± 0.53	2.22 ± 0.52	0.67	2.3 ± 0.54	2.0 ± 0.69	0.03^*^
Emotional eating	2.3 ± 0.8	2.57 ± 0.8	0.07	2.1 ± 0.78	2.3 ± 0.85	0.26	2.5 ± 0.92	2.1 ± 0.67	0.24	2.5 ± 0.76	2.9 ± 0.65	0.006^*^
Uncontrolled eating	2.43 ± 0.55	2.48 ± 0.61	0.33	2.42 ± 0.56	2.26 ± 0.6	0.28	2.4 ± 0.5	2.2 ± 0.57	0.33	2.4 ± 0.55	2.8 ± 0.46	0.006^*^
RVFQ												
Food choice (%)	16.4 ± 20.8	22.9 ± 24.7	0.02^*^	14.2 ± 17.2	20.4 ± 23.6	0.14	19.4 ± 29.3	16.6 ± 17.1	0.75^*^	17.6 ± 21.5	27.9 ± 27.8	0.05
24 hours recall												
Energy intake (Kcal)	1665 ± 386	1512 ± 395	0.007^*^	1628 ± 354	1493 ± 366	0.1	1654 ± 400	1413 ± 252.3	0.1	1701 ± 414	1567 ± 466	0.26
Protein intake (g)	66.1 ± 20.5	58.1 ± 20.3	0.006^*^	62.7 ± 15.8	55.3 ± 17.7	0.03^*^	67.7 ± 27.4	60.9 ± 19.9	0.65	68.8 ± 22	60.8 ± 23.5	0.12
Carbohydrates (g)	218.1 ± 61.3	208.5 ± 56.6	0.25	213.5 ± 61.3	206.5 ± 56.5	0.52	240.7 ± 66.8	207 ± 45.0	0.27	216.7 ± 59.7	211.2 ± 61.7	0.68
Fiber (g)	19.7 ± 7.8	19.5 ± 7.6	0.95	19.3 ± 7.1	20.4 ± 8.43	0.46	20.9 ± 7.6	18.6 ± 7.2	0.61	19.9 ± 8.6	18.6 ± 6.9	0.59
Lipids (g)	58.5 ± 21.2	49.6 ± 20.5	0.004^*^	57.3 ± 18.7	49.6 ± 18.3	0.05	48.7 ± 24.3	38.7 ± 15.4	0.35	62.0 ± 22.1	53.2 ± 23.8	0.1
SAFA (g)	18.3 ± 7.7	15.0 ± 6.9	0.002^*^	18.0 ± 6.8	15.2 ± 6.6	0.03^*^	16.1 ± 10.1	13.1 ± 4.9	0.69	19.3 ± 7.7	15.6 ± 7.9	0.05
MUFA (g)	8.65 ± 5.52	6.41 ± 4.8	0.002^*^	9.3 ± 4.9	5.9 ± 4.5	0.01^*^	5.8 ± 4.6	4.8 ± 4.2	0.65	8.7 6 ± 6.0	7.4 ± 5.3	0.28
PUFA (g)	4.6 ± 3.7	2.99 ± 2.7	0.0001^*^	5.0 ± 3.3	2.8 ± 2.4	0.01^*^	2.8 ± 2.5	1.89 ± 2.5	0.35	4.72 ± 4.1	3.4 ± 3.1	0.21
Trans	0.75 ± 0.8	0.43 ± 0.49	0.001^*^	0.88 ± 0.88	0.38 ± 0.4	0.01^*^	0.58 ± 0.67	0.4 ± 0.65	0.24	0.67 ± 0.7	0.49 ± 0.55	0.19
Cholesterol (mg)	133.2 ± 67.9	106.2 ± 62.7	0.01^*^	125.9 ± 61.0	95.7 ± 49.6	0.02^*^	116.0 ± 80.3	95.1 ± 82.9	0.53	144.2 ± 69.9	122.9 ± 68.9	0.25
w3 (mg)	0.55 ± 0.54	0.35 ± 0.36	0.007^*^	0.58 ± 0.4	0.4 ± 0.3	0.06	0.26 ± 0.32	0.27 ± 0.38	0.8	0.6 ± 0.6	0.3 ± 0.3	0.04^*^
W6 (mg)	3.27 ± 3.3	2.0 ± 2.2	0.005^*^	3.44 ± 2.9	1.9 ± 1.9	0.01^*^	1.59 ± 2.2	1.1 ± 2.1	0.57	3.52 ± 3.7	2.4 ± 2.5	0.14
Iron (mg)	8.01 ± 4.1	7.38 ± 4.12	0.4	8.1 ± 4.1	8.0 ± 4.29	0.9	8.9 ± 5.7	8.9 ± 4.6	0.92	7.6 ± 3.7	6.0 ± 3.4	0.1
Food addiction criteria	2.3 ± 1.5	2.58 ± 2.01	0.9	1.91 ± 1.0	1.86 ± 1.7	0.33	2.0 ± 1.1	1.67 ± 1.2	0.39	2.9 ± 1.88	3.7 ± 2.0	0.06

* Significant differences were analyzed with the nonparametric Mann–Whitney by MLGP group.

*p < 0.05; **p < 0.001.

When we categorize by nutritional condition, we saw that in the Normal weight subjects the MLGS ≥2.0 group showed higher scores of cognitive restriction (ns), and lower intake of protein, SAFA, MUFA, PUFA, dietary cholesterol, omega-3 and Omega-6 fatty acids (*p* < 0.05). No differences were observed in the overweight group. Finally in the subjects with obesity we found lower scores of cognitive restriction and higher scores of Emotional eating, Uncontrolled eating (*p* < 0.05) and % of food choice in the RVFQ (*p* = 0.05). In the 24-h recall we found a lower omega-3 fatty acids intake (*p* < 0.05).

### Relation between MLGS score and food addiction

No difference was observed in the frequency of diagnosis of food addiction by categories of MLGS (MLGS <2.0 and MLGS ≥2.0), in the total sample both genders, and by gender. Stratified by nutritional condition it was observed that in participants with obesity a greater % of food addiction was found in the group scored “MLGS ≥2.0 or low dopamine signaling” (53%), compared to the group scored “MLGS <2.0 or high dopamine signaling” (23%) (*p*-value; 0.05).

In the entire sample we did not find a significant correlation between MLGS, anthropometric and eating behavior variables. When the sample was categorized by nutritional condition, in the normal weight group we observed a positive and significant association between Emotional Eating and Uncontrolled Eating scores. A positive correlation was found between MLGS and Emotional Eating scores in the participants with obesity (*r* = 0.21; *p* < 0.05). In females there was a nearly significant positive association between MLGS and % food choice (*p* = 0.05).

## Discussion

The present study evaluate the association between the multilocus genetic score, with anthropometric measurements, eating behavior, food reinforcement and food addiction (FA), in a population of adults from Chile. In our results we observed that 0.98% of the sample did not carrier the risk alleles and 61% had MLGS of two or higher.

According to MLGS, we did not find any differences in anthropometric measurements. These results are in agree with the study of Romer et al., who reported the BMI was not significantly associated with polygenic scores in adults (30), and also with the study conducted in Malaysian university students, which found that three SNPs (rs1800497, rs1079597, rs1800498) in DRD2 are not associated with obesity or adiposity (31). Nevertheless, this is in contrast to the results of Yokum et al. who reported an association with high DA signaling and future weight gain, reflecting that a high DA signaling promt increases in BMI, and with a longitudinal study that showed that the C- allele of the DRD2 rs6277 exhibits protective effects on weight gain (32).

In eating behavior, we observed that participants scoring MLGS ≥2.0 (*Low dopamine signaling*) had a higher % of food choice in the RVFQ, whereas subjects in the obesity group exhibited lower cognitive restriction scores and higher emotional eating, uncontrolled eating, and percentage of food choice.

In relation to eating behavior and obesity, an important theoretical background have related to the Reward Deficiency Syndrome, which emphasizes the neurofunctional parallels among pathological eating and drug addiction. The theory describes a hyposensitive reward system that motivates an individual to overeat to increase brain DA as a form of “self-medication” due to a hypodopaminergic trait (33, 34). Particularly, contrary to the idea of overfeeding to reestablish low concentrations of brain DA, an alternative thesis led to the Reward Surfeit Model. This indicates that persons with obesity are more reactive to food rewards, resulting in increased sensitivity to rewards (17, 35).

Based on our results, subjects scoring MLGS ≥2.0 (*Low dopamine signaling*) displayed no differences in their eating behavior scores on the TFEQ in the entire sample, but higher % of snack food choice on the RVFQ, reflecting a higher relative reinforcing value of snack food. Also we observed in subjects with obesity that scored in the MLGS ≥2.0, lower scores of Cognitive Restriction and higher scores of Emotional eating, Uncontrolled eating and % of snack food choice. Also the MLGS correlated positively with Emotional Eating scores (*r* = 0.21) and % food choice in female. This results are in accordance with Stice et al. who described that individuals with a higher number of these genotypes showed a lower level of activation in reward regions, such as the putamen, caudate, and insula, in response to monetary rewards, suggesting that individuals who have a greater number of variants associated with low DA signaling may perceive food rewards and monetary rewards as more important (29). And also with the results of Diekhof et al., who demonstrated that reward-related activation in the ventral striatum and ventral tegmental area (VTA) was significantly modulated by biologically informed MLGS profiles and sex (36). In relation to the overconsumption of drugs of abuse or palatable food, considering their reinforcing properties it has been described that the 7-repeat (7R) allele of a number of tandem repeats (VNTR) in DRD4, appears as a contributing factor in the neurobiological mechanisms underlying drug abuse, aberrant eating behaviors and related comorbidities (37). Also, the literature have report some longitudinal data. In this sense Fontana et al., developed a prospective cohort study in 359 children recruited at birth. They assessed the relation between genetic variants of dopamine genes such as the DRD4 (exon 3 VNTR) and weight observing that in the first year of life, DRD4.7R variant showed higher BMI Z-scores, and at 3–4 years of life a higher intake of palatable foods and a waist circumference, suggesting that carriers of these alleles can present an increased risk for obesity related to overeating.

In relation to addiction, we found no difference in the frequency of food addiction by categories of MLGS (MLGS <2.0 and MLGS ≥2.0) in the total sample, but in the group with obesity a greater percentage of food addiction was found “MLGS ≥2.0 or *low dopamine signaling*” (53%) vs. “MLGS <2.0 or *high dopamine signaling*” (23%). These results are somewhat in line with the results of Steiger et al., who studied the relation between dopamine genetic variations (DRD2 Taq1A, DRD4 7R, and COMT) and the risk of substance abuse in women with binge-purge eating syndromes. It was shown that women who carried high function COMT and low-function DRD4 7R alleles (higher risk) showed more substance abuse (cannabis) (38). Another study in a large cohort of Italian patients with eating disorders has suggested that the specific combination of variants in DRD2 and DRD4 genes are predisposing factors for EDs.

This contrast with previous research, examining the relationship between MLGS and food addiction, supporting that, high dopaminergic signaling genotypes are linked to obesity and higher food addiction scores, through the mechanism of higher responsiveness to eating (17, 30, 39).

Several genotypes isolated are associated with putatively low DA signaling. Individuals with an A1 allele instead of an A2/A2 allele of the TaqIA polymorphism and individuals with an Ins/Ins genotype instead of a Del-allele of the DRD2-141C Ins/Del polymorphism have fewer D2 receptors (40). In this sense, it has been suggest that altered availability of dopamine receptors specifically DA2/3R in extra-striatal and dopamine cell bodies may constitute biological vulnerability traits, for addictions (41).

Stice et al. showed that a lower caudate response predicted body fat gain in adolescents carrying TaqI A1 allele (less dopamine signaling) (42, 43). Cohen et al. showed that TaqIA A1 allele carriers have lower activation of the orbitofrontal cortex (OFC), amygdala, and hippocampal areas to monetary rewards (44) and lower activation in the midbrain, thalamus, and OFC to food rewards (45). Also the single nucleotide exchange in the Catechol-O-methyltransferase gene (COMT Val158Met), have shown fourfold less COMT activity in Met homozygotes compared to Val homozygotes (46) and according to Lachman et al. (47), the former have higher levels of tonic DA and less phasic release in the striatum.

Recently, Arrue et al. explored the relationship of cardiometabolic alterations with single genetic polymorphisms DRD2 in 285 psychiatric patients, they showed that a low dopaminergic activity was related to higher risk of suffering obesity, high diastolic blood pressure (DBP), and hypertriglyceridemia (HTG) (48). Also, Silveira et al. recently showed that variations in a MLGS reflecting DA signaling, was associated with differences in sugar intake in Children that had intrauterine growth restriction, suggesting that DA function is involved in this behavioral feature in these children (49).

This study has several limitations and strength. The fact that the data did not support our hypotheses about food addiction and MLGS could be explained in part by some methodological issues. (i) The small sample size of our study was obtained based on convenience and cannot generalized to all Chilean adults; (ii) Our results could be limited due to the small number of adults who met the criteria for food addiction; (iii) We evaluate dietary intake using 24-h recalls that have document several bias and could be responsible of some inconsistency in our results showing higher food choice, but lower energy and macronutrient intake by MLGS. Additionally, one limitation of our study was that we did not assess anxiety and depression levels in our sample, despite the fact that some studies have shown that anxiety, depression, and emotional eating are closely related (50, 51). This limitation could result in biases and misinterpretations of our results. Nevertheless, this study has the following strengths: (i) This is the first study in Chilean population that considers a multilocus approach, increasing the small contribution of individual polymorphisms to phenotypic variance; (ii) In order to measure eating behavior, we utilized a wide range of tools that were measured face-to-face by highly trained dietitians.

We conclude that, although we did not find any relationship between food addiction and MLGS, these results provide evidence for the involvement of genotypes associated with low dopaminergic signaling in eating behavior, specifically in snack food choice, emotional eating, and uncontrolled eating. These findings strongly encourage further investigations related to genetic susceptibility and the risk of chronic overeating, including the possibility to explore other pathways related to dopamine, such as physical activity. Dopamine is known to regulate physical activity, and in general studies reported in the literature as ours, do not consider this variable, open to the question of whether reduced D2R disrupts energy expenditure and activity. It is frequently suggest that reductions in D2R commonly create a reward deficit and altered appetitive motivation, which induce compulsive eating and obesity. Nevertheless, Beeler et al. developed a D2R knockdown (KD) mouse line and assessed energy expenditure and appetitive motivation under conditions of diet-induced obesity. Interestingly, the KD mice did not gain more weight or showed increased appetitive motivation and in an enriched environment with voluntary exercise opportunities, exhibited dramatically lower activity and became more obese than wild-type mice (52).

## Data Availability

The raw data supporting the conclusions of this article will be made available by the authors. Requests can be directed to the corresponding author.

## Footnotes

^1^ http://www.uss.cl
